# Apologizing for Risque Stories

**Published:** 1917-06

**Authors:** 


### Apologizing for Risque Stories

It seems that some of the physicians who indulge in suggestive stories at medical banquets think that the medical profession as a whole appreciate such practices, but that it is proper to apologize to the TEXAS MEDICAL JOURNAL for them. Fortunately, the TEXAS MEDICAL JOURNAL does not have to listen to them, so the apologies should be made to the physicians present whose refinement does not allow them to enjoy such things. The following letter has just been received from a prominent physicion of that class, and expresses the disgust held by many for dirty banquet speeches:

"I always read the JOURNAL with interest, and especially those short and efficient articles that leave out the wind, text-books, and theories and get right down to the everyday, busy doctor's style of writing. I am now writing to ask you to stand by your guns in re smutty yarns at smokers and banquets. I have just read on page 220 of April *American Medicine* an idealistic diatribe upon the purity of mind of doctors, and calling upon you to offer apologies to the profession for your onslaught. *American Medicine* wrote as he knew it should, could, and would be, but just simply forgot. Even at our last State medical meeting, after your request was published, our smutty yarners did their supposedly witty stunts 'with apologies to the TEXAS MEDICAL JOURNAL.' They seemed to delight in seeing how near they could come to being vulgar without coming right out and saying shocking things, by innuendo making it all too clear the unsaid meanings, thus rivaling Shakespeare in cleverly covering naked smut.

"I think a few more articles placed so timely will bear better fruit until all rottenness will be eliminated from our smokers."

